# A Framework for Flood Risk Analysis and Benefit Assessment of Flood Control Measures in Urban Areas

**DOI:** 10.3390/ijerph13080787

**Published:** 2016-08-05

**Authors:** Chaochao Li, Xiaotao Cheng, Na Li, Xiaohe Du, Qian Yu, Guangyuan Kan

**Affiliations:** 1 State Key Laboratory of Simulation and Regulation of Water Cycle in River Basin, Research Center on Flood & Drought Disaster Reduction of the Ministry of Water Resources, China Institute of Water Resources and Hydropower Research, Beijing 100038, China; chengxt@iwhr.com (X.C.); lina@iwhr.com (N.L.); duxh@iwhr.com (X.D.); yqcherie@126.com (Q.Y.); kanguangyuan@126.com (G.K.); 2 College of Water Conservancy and Hydropower Engineering, Hohai University, Nanjing 210098, China

**Keywords:** flood risk analysis, flood control measures, UFSM, UFDAM, R-D function, EAD (expected annual damage)

## Abstract

Flood risk analysis is more complex in urban areas than that in rural areas because of their closely packed buildings, different kinds of land uses, and large number of flood control works and drainage systems. The purpose of this paper is to propose a practical framework for flood risk analysis and benefit assessment of flood control measures in urban areas. Based on the concept of disaster risk triangle (hazard, vulnerability and exposure), a comprehensive analysis method and a general procedure were proposed for urban flood risk analysis. Urban Flood Simulation Model (UFSM) and Urban Flood Damage Assessment Model (UFDAM) were integrated to estimate the flood risk in the Pudong flood protection area (Shanghai, China). S-shaped functions were adopted to represent flood return period and damage (R-D) curves. The study results show that flood control works could significantly reduce the flood risk within the 66-year flood return period and the flood risk was reduced by 15.59%. However, the flood risk was only reduced by 7.06% when the flood return period exceeded 66-years. Hence, it is difficult to meet the increasing demands for flood control solely relying on structural measures. The R-D function is suitable to describe the changes of flood control capacity. This frame work can assess the flood risk reduction due to flood control measures, and provide crucial information for strategy development and planning adaptation.

## 1. Introduction

**Figure 1:**
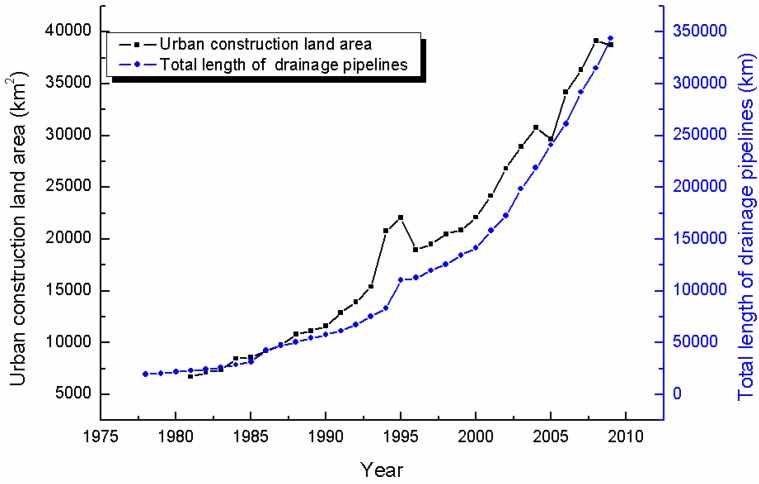
Statistics of total length of drainage pipelines and urban construction area.

With the rapid development of urbanization, flood risks become more and more severe [1]. Since the 1950s, the number of urban flood disasters has been gradually rising in China. The flood damage is higher than it was in the past. According to the 2015 China flood and drought report, more than 100 cities have suffered waterlogging per year since 2006. The numbers of waterlogged cities were 130 in 2008, 258 in 2010, and 234 in 2013, respectively. Nearly 62% of 351 cities were waterlogged between 2008 and 2010. The vast majority of flooding and waterlogging disasters are caused by local extreme rainstorms. Flooding and waterlogging disasters remain one of the main challenges faced by developing counties. They not only cause high mortality and suffering, but also damage local economies that are in process of formation and thwart development achievements [2]. To ensure security, a large number of flood control works and drainage systems have been built to reduce the economic losses associated with flood disasters. As shown in Figure 1, the total length of drainage pipelines is proportional to urban construction land area, and the correlation coefficient is 0.96.

For flood hazard-affected bodies themselves, the subjective reasons for frequent occurrence of flooding and waterlogging disasters are analyzed as follows: (1) Cities expand in high flood hazard areas; (2) The development of drainage systems is slower than the city development; (3) Impervious areas increase; (4) The large population and property density result in an increased flood vulnerability in urban areas. 

With the continuous expansion of cities, the urban flooding and waterlogging problems are expected to become worse and worse unless more effective measures are adopted. The flood control work measures can be divided into two types: structural and non-structural. Key elements of structural works have been reservoirs, dikes, detention basins, pumping stations, etc. Non-structural measures include flood forecasting, flood emergency planning and response, and post-flood recovery. Distinct from technical support services, these activities directly modify the vulnerability of communities exposed to flood risks. Typically protection measures such as dikes, levees, seawalls have a certain designed capacity. When the flood scale exceeds this capacity, the structural measures could fail, and the flood damage could be more disastrous. The “protection” of flood control systems provides perverse incentives for private individuals and businesses to adapt on their own devices and may promote unwanted concentrations of population/businesses in hazard-prone areas. Thus it is difficult to meet the increasing demands for flood control solely relying on structural measures. Appropriate non-structural measures provide sound strategies for sustainable development. According to Wang’s research, reasonable land use planning based on flood risk analysis will decrease the future flood risk by 39%–50% in Taihu Basin [3]. The economic losses associated with flood and waterlogging can be reduced by scientific flood risk management. Reasonable flood risk analysis can provide crucial information for strategy development and planning adaptation.

Natural disaster risk assessment methods generally are of three types: mathematical statistical methods, index system methods, and dynamic risk evaluation methods based on integrated models. The disaster trends are summarized based on the first method according to the analysis of historical flood disaster data. Benito provided a scientific method based on palaeoflood and historical data for flood risk analysis [4]. The index system methods focus on the selection of disaster risk indexes, optimization, and calculation of their weights. Okazawa established a global flood risk index based on both natural and social factors [5]. In order to estimate the flood risk index at a spatial resolution of city/county/town units for a river watershed, Seiler used the standardized precipitation index for flood risk monitoring [6]. With the development of hydrological models, hydraulic models and Geographic Information System (GIS) technology, integrated models have been widely used in recent years. Flood inundation information and socio-economic information are analyzed by spatial overlay analysis [7,8]. Detailed geographic information data is needed to construct integrated models based on GIS. For the first two methods, it is difficult to describe the relationship among the main factors, or provide the evolutionary trends of flood risk and time-series inundation information. When the evaluation objects and conditions change, these methods cannot adjust in real time and the uncertainty and dynamics of the disaster system cannot be revealed as well, so the third method has become the mainstream direction of current research on natural disaster risk assessment.

Crichton proposed that flood risks depend on three elements: hazard, vulnerability, and exposure [9]. The flood risk can then be represented as follows [10,11]:

(1) flood risk=function (hazard, exposure, vulnerability) 

The hazard means the threatening natural event including its probability of occurrence, and it generally quantified as the water depth and water flow velocity distribution. The exposure refers the people/assets that are present at the location involved. The vulnerability indicates the characteristics and circumstances of a community, system or asset that make it susceptible to the damaging effects of a hazard [12]. Flood disasters are predictable and controllable, which are features that distinguish floods from other natural disasters such as earthquakes and volcanic eruptions. The capacities of flood forecasting, early warming and flood control can be enhanced to mitigate the impact flood disasters. Hence, the capacity should be also considered. The capacity can be quantified as the flood risk reduction. The flood risk reduction comprises the flood damage averted in the future as a result of schemes to reduce the frequency of flooding or reduce the impact of that flooding on the affected property and economic activity, or a combination thereof. Spatially explicit hydrodynamic flood simulation models play an important role in flood risk reduction assessment.

However, flood risk assessment in urban areas is more complex than in rural areas because of their closely packed buildings, different kinds of land uses, and large amounts of flood control works and drainage systems [13]. In this study, a framework was developed which combines urban flood simulation and flood damage assessment. A key element of this framework that makes it suitable for risk reduction assessment is the ability to provide objective inundation information with the consideration of buildings, land uses and flood control works. In terms of the concept of the risk triangle, the hazard, exposure, and vulnerability of storm waterlogging were analyzed. The R-D function was proposed to measure the effectiveness of flood control works and provide indications of the changing resilience. The results could be critical for land use planning, for flood control works design, for mapping evacuation egress routes, and for locating suitable emergency shelters to name but a few risk treatments [14].

## 2. Study Area

**Figure 2:**
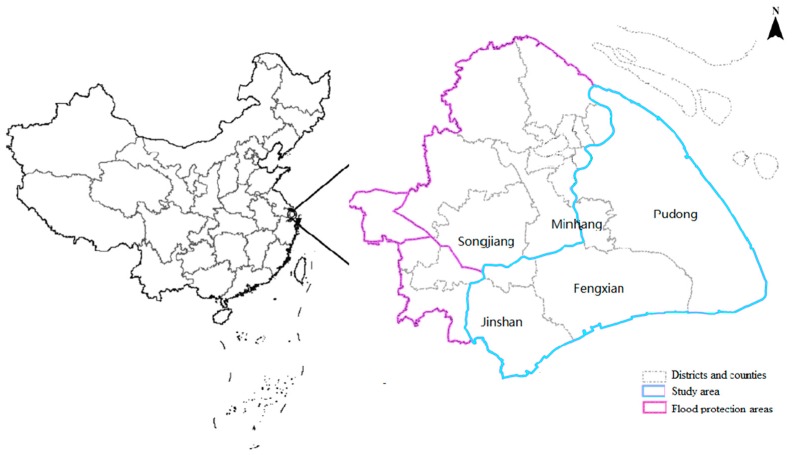
The location of the Pudong flood protection area.

Shanghai, which is located in the estuary areas of the Yangtze River, is one of the most important core regions of economic, transportation, industry, science and technology in China (Figure 2). Shanghai has developed rapidly due to its various advantages, such as a highly developed industry, a stable economic base, and a dense population. Shanghai ranked first among Chinese cities in terms of GDP in 2015. Meanwhile, Shanghai is a flat land, which is prone to serious flood disasters caused by “plum rains”, typhoons, and storm surges. The flood risk features in Shanghai are very sensitive to both disaster-causing factors and social economy. Shanghai is divided into four flood protection areas: Pudong, Puxi, Hangjiahu and Yangchengdianmao. The Pudong flood protection area (2722 km^2^, hereinafter referred to as Pudong) is a coastal part of Shanghai located in the right bank of the Huangpu River, which is taken as the study area in this paper. The estuary of the Yangtze River is situated to the north, the East China Sea to the east, and Hangzhou Bay to the south. The location of Pudong is shown in Figure 2. The Huangpu River originates from Tai Lake and Dianshan Lake. It meanders through the whole city from west to east in the upstream. The terrain of Pudong is relatively flat. The ground surface elevation ranges from 3.5 m to 4.5 m based on the Wusong Datum, and the northwest is lower than the southeast. The Pudong flood protection area contains five administrative districts: Pudong New District, Fengxian District, part of Minhang District, part of Songjiang District, and part of Jinsan District. Considering the threat of flooding caused by typhoons, plum rains and river floods, Shanghai has implemented a comprehensive flood defense system which consists of dikes, floodwalls, gates, pumps and drainage pipe networks.

Shanghai is vulnerable to flooding due to its geographic location on flat and low-lying terrain, rapid socio-economic development, land subsidence, and climate change. In 2005, No. 9 Typhoon Matsa affected Shanghai. It caused a direct economic loss of 1.358 billion CNY, which included agricultural losses of 843 million CNY and industrial losses of 158 million CNY. A recent flood occurred in 2013, caused by No. 23 Typhoon Fitow. It was reported that several river dikes in the upstream were overtopped or broken, and the maximum inundation depth in Songjiang District was estimated at more than 2.5 m [15,16,17]. The adjacent farmland and residential areas were flooded.

## 3. Methodology

### 3.1. Framework of Flood Risk Analysis

**Figure 3:**
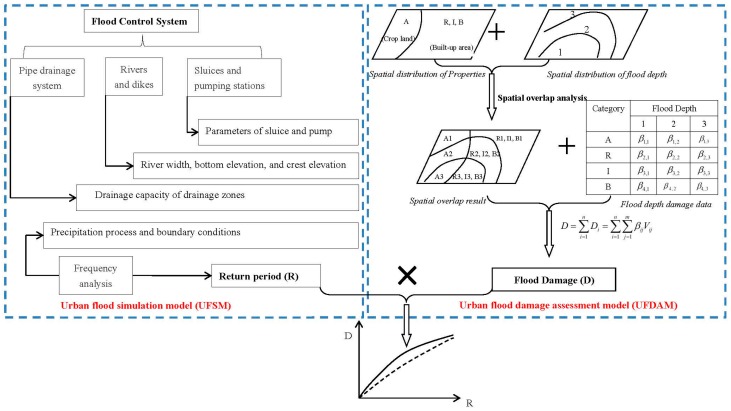
A basic framework of flood risk analysis.

Urban Flood Simulation Model (UFSM) and Urban Flood Damage Assessment Model (UFDAM) are developed by China Institute of Water Resources and Hydropower Research (IWHR). The UFSM and UFDAM are coupled to evaluate the flood risk in this study. The flood risk analysis framework and benefit assessment method of flood control measures are adopted in this paper. The methodology contains a number of steps (Figure 3):

1. Flood and waterlogging scenarios are simulated by UFSM. Several model parameters are set up corresponding to the real operation of flood control works.
2. Flood damage is evaluated by UFDAM. The index system of flood damage assessment is designed according to the indicators of national statistics data.
3. Flood damage curve is constructed based on S-shaped function. The return period-damage function is selected to describe the flood risk.

#### 3.1.1. UFSM

**Figure 4:**
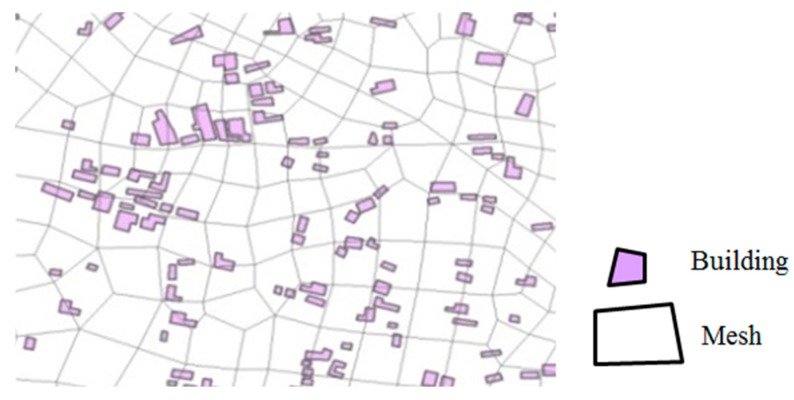
The distribution of residential buildings and the generated mesh.

UFSM is a 1D–2D coupled hydrodynamic model which has been widely used for flood and waterlogging simulation in urban areas. The approach adopted in this study includes modelling of the river channel and main road as a 1D network nested within the 2D domain representing the floodplain. It is necessary to consider the impact of buildings, land use and flood control works on flood risk analysis. The residential buildings are densely distributed in urban areas. These buildings would block and change the direction of water flow. As Shown in Figure 4, the building spaces should be deducted when we calculate the inundation areas. Thus, a parameter, adjusted area rate (AAR), is adopted in this model, which can be calculated with Equation (2).

(2) $$AAR=1-\frac{A_{b}}{A_{m}}$$

where *AAR* is the adjusted area rate of a mesh; $A_{b}$ is the area of building within the mesh; and $A_{m}$ is the area of the mesh.

Different land use types have different impacts on the runoff process. Parameters related to the runoff process should be set up according to the land use, such as runoff coefficients and roughness. The initial values of the runoff coefficient and roughness can be given according to the reference values. The reference values of roughness for different land use are proposed in Table 1. The parameters can be adjusted and identified by comparing simulation results with measured results.

The runoff coefficient of impervious areas is about 0.9, and the runoff coefficient of natural catchments is about 0.5. The runoff coefficient can be calculated by linear interpolation according to the impervious area ratio. The impervious area ratio approximates the *AAR*. Thus:

(3) R=0.5+(0.9−0.5)×AAR

where *R* is the runoff coefficient.

The flood control works in urban areas generally contain dikes, pumps, sluices, and pipe drainage networks. When the water level of a river is lower than the crest elevation, there is no water flow exchange between the river and the floodplain. When the water level exceeds the crest elevation or a dike breaks, the weir formula is used to calculate the flow. Gates and pumps can be simulated according to the operation rules. Drainage pipe networks are simplified as several underground reservoirs. Details of UFSM can be found in Cheng [18].

#### 3.1.2. UFDAM

**Figure 5:**
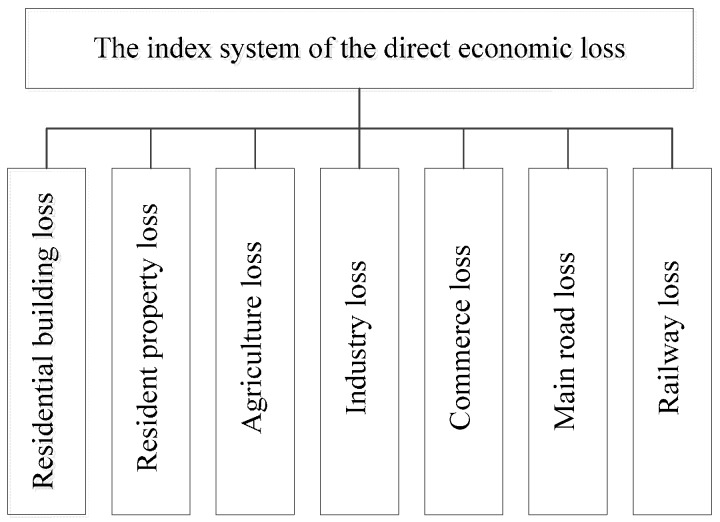
The index system of the direct economic losses.

The flood damage assessment method is combined with GIS technology. The direct economic losses can be estimated according to the loss ratios of different kinds of hazard-affected bodies. The hazard-affected bodies are divided into several categories: residential buildings, residential properties, industry, commerce, agriculture and transportation. The residential building losses refer to the cost of rebuilding the structure-damaged and landscape-damaged buildings. The residential property losses refer to the cost of replacing furniture and household appliances such as TVs, washing machines and refrigerators. The total direct economic losses of the affected area are equal to cumulative losses of all categories. The basic social economic data can be acquired from statistics yearbooks. The flood damage assessment index system is shown in Figure 5. More details on UFDAM can be found in Wang [19,20].

#### 3.1.3. Flood Damage Function

The formulation of the flood damage function must reflect the interaction between the probability of occurrence of a hazardous event and its estimated consequences [21,22]. In recent years, a widely accepted concept of flood risk in a particular region is often termed as expected annual damages (EAD) [23,24,25]. EAD is a cost-based measurement representing the expected average infrastructure costs as a result of flooding each year, which is the integration of the complete range of all possible flood events. It can be expressed as:

(4) $$Risk=\;\int_{0}^{1}D\left(p\right)dp$$

where *D* is the damage of a given flood event, and *p* is the probability of this flood event within a year. To calculate the risk (EAD) value one needs to determine the probability distribution of flood events, the flood depth in an event, and the damages under the given depth. In this study, a practical approach is to build a damage probability curve based on different return periods of floods. The return period R is used instead of probability *p*, *p* = 1/*R*, *p* ∈ (0,1), *R* ∈ (0,∞) and then the risk can be expressed as:

(5) $$Risk=\;\int_{0}^{\infty }D\left(R\right)dR$$

**Figure 6:**
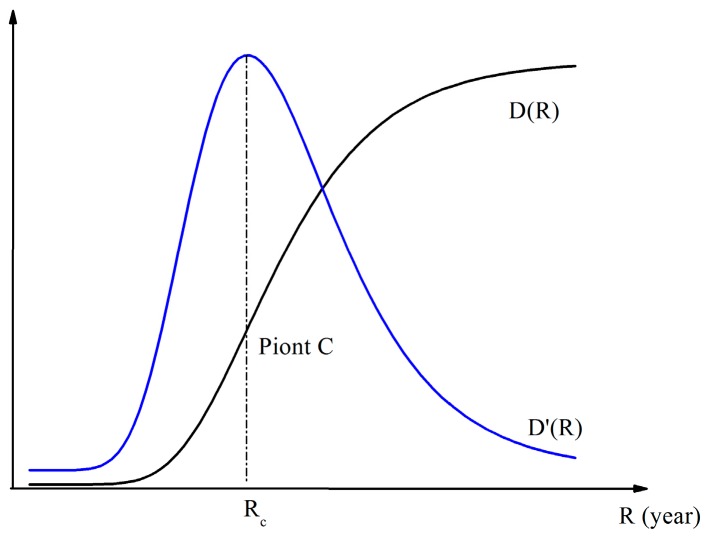
Flood R-D function curve and the first derivative curve of R-D function.

*D(R)* can be expressed as an S-shaped function according to previous studies [26,27,28]. *R* is the independent variable, and *D* is the dependent variable. As shown in Figure 6, the flood R-D curve can be expressed as an S-shaped function. The characteristics of *D(R)* function are summarized as follows: (1) the function *D(R)* increases monotonically; (2) Point C is the inflexion point where the rate of change of damage begins to decline. It is called the damage transition point. The first derivative function *D’(R)* indicates that the slope of the flood damage curve is maximum when *R* = *Rc*.

The maximum value of flood damage *A*, critical return period *Rc*, and integrated loss coefficient *k* are the three main parameters, which can be determined through experiential judgement, experimental simulation, and curve fitting. The maximum flood damage *A* is related to flood exposure such as socio-economic factors. The critical return period *Rc* can reflect flood control capability. The integrated loss coefficient *k* is used to describe flood vulnerability. The historical data or simulation data can be utilized for curve fitting. Mathematical statistical analysis tools are used to determine the values of the parameters in this study [29].

**Figure 7:**
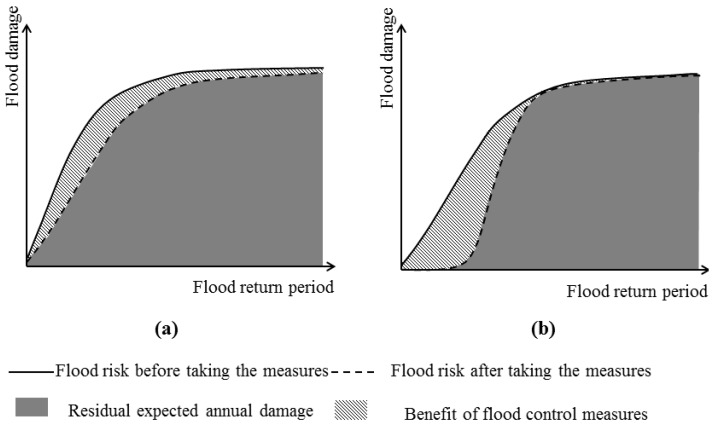
The benefit of flood control measures may have two situations: (**a**) the flood risk can be reduced under any return period of flood; (**b**) the flood risk only can be significantly reduced within the flood control capacity.

Figure 7 provides the classic diagram of calculating the benefit of flood control measures. The annual average flood damage is the area under the graph of flood losses plotted against the return period in years.

(6) $$EAB=\frac{EAD_{b}-EAD_{a}}{EAD_{b}}=\frac{\int_{0}^{\infty }D_{b}(R)dR-\int_{0}^{\infty }D_{a}(R)dR}{\int_{0}^{\infty }D_{b}(R)dR}$$

*EAB* is the benefit of flood control measures, *EAD_b_* is the flood risk before taking the measures, *EAD_a_* is the flood risk after taking the measures, *D_b_(R)* is the flood damage function curve before taking the measures, and *D_a_(R)* is the flood damage function curve after taking the measures [4].

### 3.2. Data

The geographic data, hydrological data, social economic data are the foundation for the establishment of a flood risk assessment system. DEM with a resolution of 5 m was generated by digitalizing the topographical feature elements. The elevation points in 1:10,000 topographic maps and other geo-information such as buildings, roads, land uses are provided by the Shanghai Administration of Surveying and Mapping (2012). The data of rivers, gates, pumps, and dikes are adopted according to the Second Shanghai Water Resources Survey (2012). The water surface elevation refers to the Wusong height datum. The social economic data is obtained from the 2014 statistical yearbooks of Pudong New District, Fengxian District, Minhang District, Songjiang District, and Jinsan District. The design rainfall and design water/tide levels are calculated according to the long term data from 1965 to 2013.

## 4. Results and Discussion

### 4.1. Model Validation

**Figure 8:**
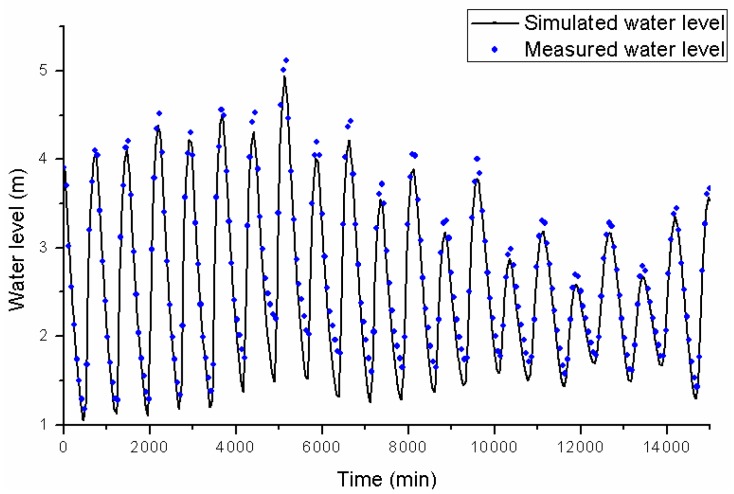
The simulation results and measured water levels of the Huangpu Park station during Typhoon Fitow.

According to the above methodology, the flood risk assessment system was established. The measured water level during Typhoon Fitow is set as the upstream and downstream boundary conditions for UFSM. A 150 m × 150 m mesh is considered appropriate in view of both the size of the river channel and computation time. The total number of meshes is 127,453. A total of 44 gates, 262 pumps, and 31 drainage division areas are simulated in the study area. Figure 8 presents the comparison between the simulation results and measured water levels at the Huangpu Park station. The simulation results show good agreement with the measured data. The simulation results are generally lower than the measured water level. The highest simulated water level is 4.94 m, the highest measured water level 5.12 m, and the relative error is 3.5%. The lowest simulated water level is 1.05 m, the lowest measured water level 1.18 m. The errors at low water level are higher than these at high water level. When the water level is low, the gates and pumps along the Huangpu River release water into the river. As the amount of water from Puxi is ignored, the simulation results are lower especially in a low tide situation.

The surveyed flood damage was 132.12 million CNY, according to the 2013 statistical data of Jinshan, Songjiang, and Qingpu District (Table 2). Through spatial overlay analyzing the flooding data and social economic data, the flood damage is calculated as 130.60 million (Table 3). The simulation result coincides quite well with the surveyed value with a relative error of 1.15%. Hence, the model can be utilized to estimate the flood damage in Shanghai.

### 4.2. Hazard Analysis

The 24-h design rainfalls with frequency of 0.001, 0.002, 0.005, 0.01, 0.02, and 0.05 were taken as input data for scenario simulation. Two flood control works conditions are set up. Condition 1: Flood control works normally operate. Condition 2: All pumps, gates and drainage systems stop working. The statistical inundation data is shown in Table 4. The benefit of established flood control works can be evaluated by comparing the difference between Condition 1 and Condition 2.

**Figure 9:**
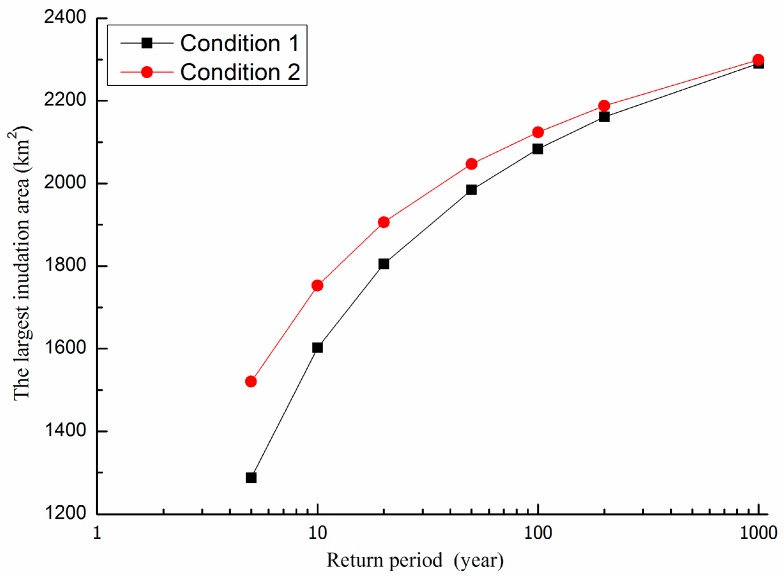
Flood inundation areas under different recurrence interval storm events.

The water depth and inundation area increase with the growth of the rainfall return period. The disaster situation of Condition 2 is more serious than Condition 1. All inundation areas are accumulated together as the largest inundation area. The largest inundation area of a 5-year flood in Condition 1 is 1287.75 km^2^, the largest inundation area of a 5-year flood in Condition 2 is 1520.35 km^2^, and the relative difference is 18%. The largest inundation area of a 1000-year flood in Condition 1 is 2290.87 km^2^, the largest inundation area of 1000-year flood in Condition 2 is 2299.33 km^2^, and the relative difference is 0.37%. The inundation areas of different return periods are compared in Figure 9.

The total inundation area increases with rainfall return period. With the increase of return period, the differences between inundation areas between Condition 1 and Condition 2 decrease. It means that the flood risk reduction due to flood control works decreases with the flood return period. The flood risk reduction effect becomes non-significant under extreme storm conditions. The inundation area is reduced by 15.76%.

**Figure 10:**
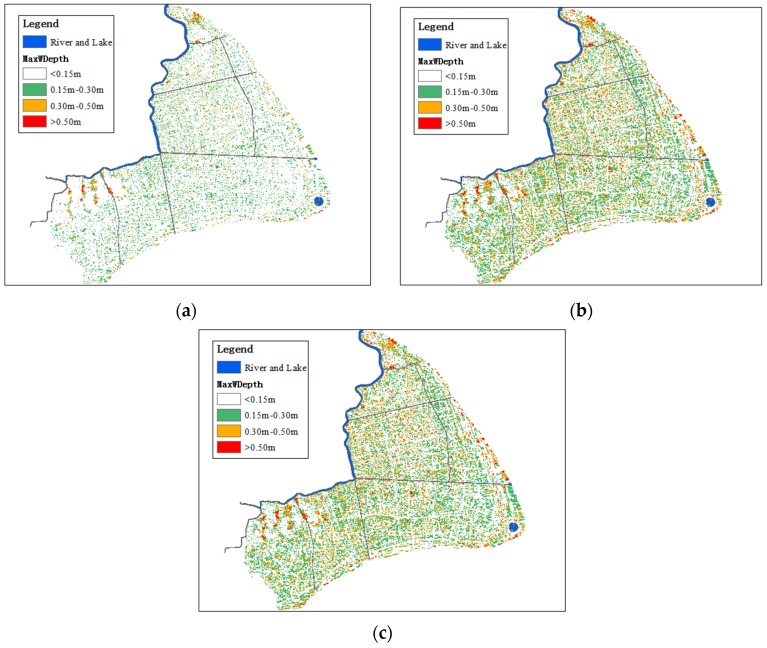
(**a**) 1 in 10 years flood hazard map; (**b**) 1 in 100 years flood hazard map; (**c**) 1 in 1000 years flood hazard map.

The maximum water depth was set as hazard index of flood disaster, which was divided into grades according to field surveys: 0 m–0.15 m, 0.15 m–0.30 m, 0.30 m–0.50 m, over 0.55 m [30]. Table 5 presents the normalization thresholds of the flood depth. The flood hazard maps are drawn according to Table 5. Under Condition 1, the flood hazard maps of 1/10, 1/100, and 1/1000 storm events are shown in Figure 10. With the increase of flood return period, the flood hazard increases. According to the flood hazard analysis, the hazard is higher in the upstream of Huangpu River and the coastal area. The terrain of Jinshan and Songjiang District is low-lying, thus the flood hazards of these areas are higher. Land reclamation projects were carried out in coastal area recently, for example, Lingang New City Project. The areas reclaimed from the sea are prone to flooding as well.

### 4.3. Vulnerability Analysis

**Figure 11:**
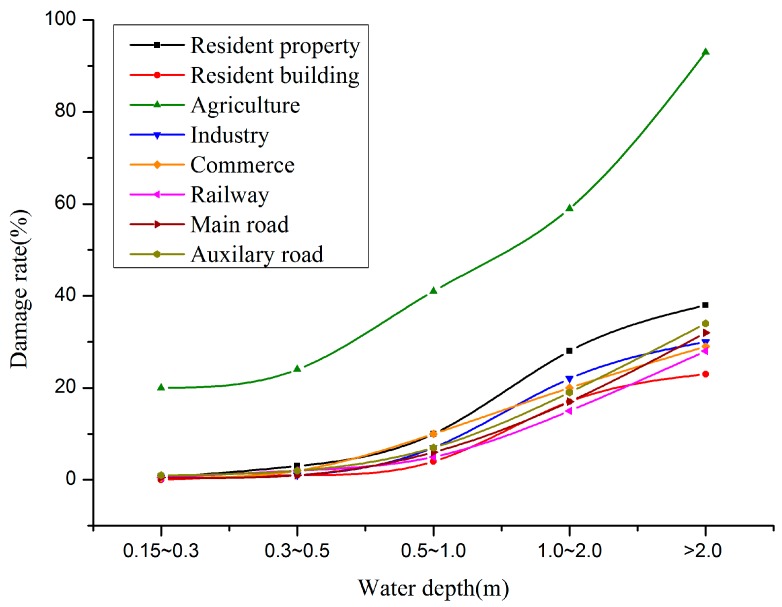
Loss rate relations of different water depth.

The damage caused by flooding is related to flooding characteristics, such as depth, velocity and duration of flooding. In this paper the damage is estimated based on the depth of flooding which is important and also relatively easy to determine. The loss rate is set up according to the research of Nanjing Institute of Geography & Limnology and the disaster survey data of typhoons and torrential rains [31]. The damage rate relations are shown in Figure 11.

### 4.4. Exposure Analysis

**Figure 12:**
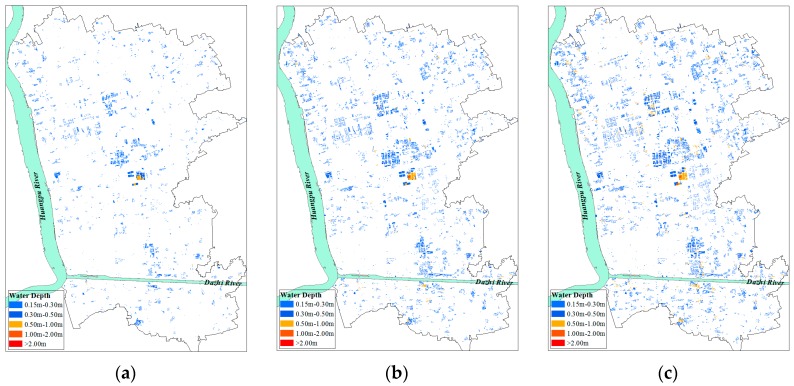
(**a**) The exposure of residential building under 1 in 10 years extreme flood; (**b**) The exposure of residential building under 1 in 100 years extreme flood; (**c**) The exposure of residential buildings under 1 in 1000 years extreme flood.

**Figure 13:**
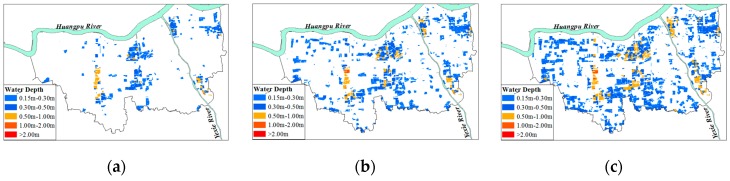
(**a**) The exposure of cultivate land under 1 in 10 years extreme flood; (**b**) The exposure of cultivate land under 1 in 100 years extreme flood; (**c**) The exposure of cultivate land under 1 in 1000 years extreme flood.

Exposure is often used to describe the people and assets that would be subjected to the threat of floods, such as population, buildings, crops and lifelines. In this study, exposure presented as the number of external structure and internal property value of residential buildings. Residential areas and land uses are extracted based on the 1:10,000 topographic map of the study area in 2012. With the increase flood return period, the flood exposure grows. The residential building density is high in Minhang District, and its exposure map is drawn in Figure 12. A large proportion of the residential buildings is exposed to the inundation water depth below 0.5 m. The cultivated land density is high in Songjiang District, and its exposure map is drawn in Figure 13. A large proportion of the cultivated lands are exposed to inundation water depths below 0.5 m. Some cultivated lands near both banks of the river are submerged at higher water depths.

**Figure 14:**
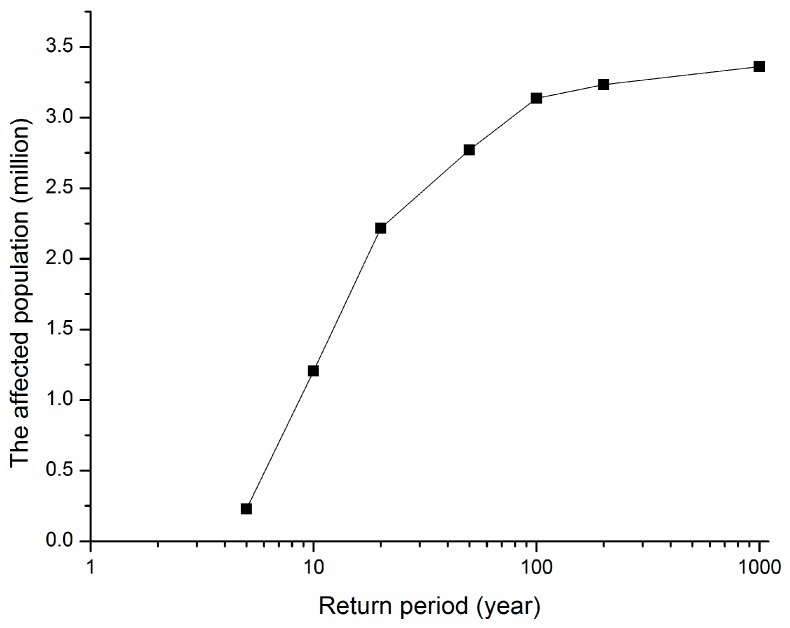
Flood affected population under different recurrence interval storm events.

We assume that the population distribution of administration district is uniform. Then the affected population can be calculated with Equation (7):

(7) $$P_{e}=\overset{n}{\underset{i=1}{\sum }}d_{i}\overset{m}{\underset{j=1}{\sum }}A_{i,j}$$

where, $P_{e}\;$is the affected population, i is the number of the administration district, n is the total number of the administration district within the study area, j is the number of the grid cell within a certain residential district, m is the total number of the grid cells within the residential district, $d_{i}$ is the population density of the administration district i, and$\;A_{i,j}$ is the inundation residential building area of the grid *j* within the administration district i. The affected population under different return periods is shown in Figure 14.

### 4.5. R-D Function Construction

The direct economic losses of the classified assets depending on flood water depth in each sub-district can be estimated separately according to the values of classified assets, relevant inundated area of flood events with different return periods and the damage rates. The flood damage result is shown in Table 6. The losses of temporary production and service cessation are ignored in this paper. These kinds of losses are related to the inundated shut-down time, so the losses of the industrial and commercial sectors are probably underestimated in this paper.

Two functions are selected to fit flood damage: Gompertz function and Logistics function.

(1) Gompertz function:
  (8) $$D=Ae^{-e^{-k\left(R-R_{c}\right)}}$$
  where, *D* is the flood damage, *R* is the return period of flood, *A* is the maximum damage, *Rc* is the critical period, and *k* is the integrated loss coefficient. Function characteristics: the minimum value is 0, the maximum value is *A*, the abscissa of critical point is *Rc*, and the maximum slope is *Ak/e*.
(2) Logistics function:
  (9) $$D=\frac{A}{1+e^{-k(R-R_{c})}}$$

**Figure 15:**
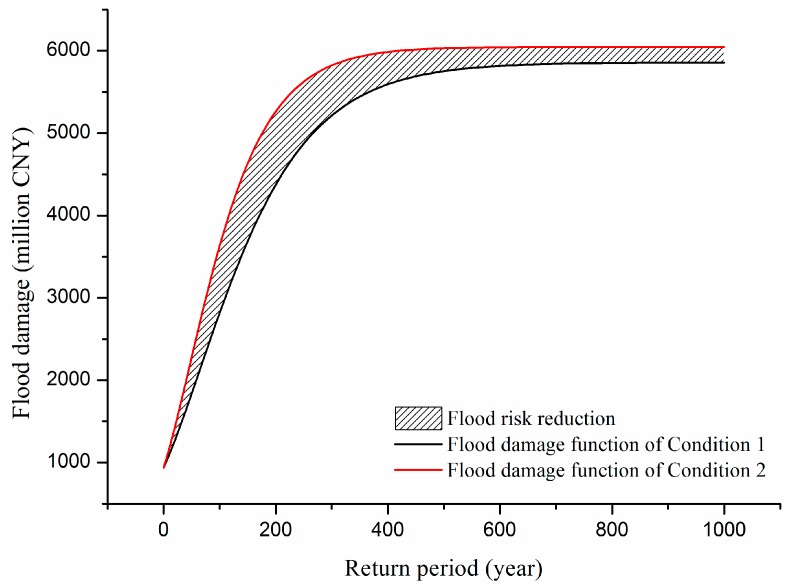
The fitting results of flood damage function.

Function characteristics: the minimum value is 0, the maximum value is *A*, the abscissa of critical point is *Rc*, and the maximum slope is *Ak/e*. The fitting results are shown in Table 7. The Adjusted *R*^2^ is particularly useful in the feature selection stage of model building. The closer that this value is to 1, the better fitting the model is. The Gompertz function is more suitable than the Logistic function to present flood damage. The critical period *Rc* of Condition 1 is bigger than Condition 2. However, the integrated loss coefficient *k* of Condition 1 is smaller than Condition 2. It means that the flood control works have an impact on both flood control capacity and flood vulnerability. The fitting results are shown in Figure 15, where the flood risk reduction is represents as the shade of gray.

Equation (6) expresses the benefit of flood control measure. It often used to assess the feasibility of new constructions. In this paper, we use it to assess the flood risk reduction due to the established flood control system. The flood risk reduction is calculated as follows:

(10) $$Risk_{reduction}=\frac{\int_{0}^{1000}D_{2}(R)dR-\int_{0}^{1000}D_{1}(R)dR}{\int_{0}^{1000}D_{2}(R)dR}=7.14\%$$

(11) $$\begin{array}{l} Risk_{reduction1}=\frac{\int_{0}^{66}D_{2}(R)dR-\int_{0}^{66}D_{1}(R)dR}{\int_{0}^{66}D_{2}(R)dR}=15.59\%\\ Risk_{reduction2}=\frac{\int_{66}^{1000}D_{2}(R)dR-\int_{66}^{1000}D_{1}(R)dR}{\int_{66}^{1000}D_{2}(R)dR}=7.06\% \end{array}$$

The damage transition point C corresponds to the parameter *Rc* (critical return period), which is associated with the integrated flood defense capability or flood control standard. *Rc* = 66.00 in Condition 1, and *Rc* = 47.97 in Condition 2. We choose the larger one. Then the benefit can be divided into two parts. Within the flood control capacity, flood risk is reduced by 15.59% and the effect of flood risk reduction is significant. Once flood magnitude exceeds the flood control standard, the flood damage will sharply increase and show the feature of mutability and the flood risk is only reduced by 7.06%.

## 5. Conclusions

Developing appropriate emergency responses to address and prevent flood inundation requires a reliable flood risk analysis including hazard, vulnerability and exposure. This paper explores the spatial distributions of flooding, the vulnerability of different kind of assets and the flood prevention capacity, particularly concerning buildings, land uses and flood control works in Pudong (Shanghai, China). A baseline framework was established that could be used to assess the flood disaster risks and flood risk reduction. We reach the following conclusions:

1. Scenario modelling of flood inundation from extreme rainfall events is a challenge, particularly in urban areas. In order to address the impact of buildings, land uses and flood control works issues on flood risk, the specific model parameters are required. The flood inundation modelling using UFSM indicated that AAR is an important parameter. It can be utilized to describe the impact of buildings and land uses. The model was validated using a historical flood event: Typhoon Fitow, which was the most serious flood in recent years. The simulation results match well with the surveyed inundation depths and inundated areas. However, the model was only tested for a single observed event. It is recommended that the model also be tested and evaluated for more storm events to enhance the reliability of the model outputs.
2. The flood hazard, vulnerability and exposure were analyzed. A flood high risk may be caused by elevation, land uses and buildings present in the area. The results indicate that the upstream of Huangpu River and the coastal area are prone to floods. The UFDAM were used in this case study. The evaluation index system of UFDAM is able to describe the indirect loss of urban disaster caused by flooding or waterlogging. These indexes should be easily adopted by the administrative department for emergency management. Hence, they are consistent with the indicators of local statistics yearbooks. The convenience of calculation and feasibility of obtaining are improved. The indirect loss should be taken in to account, and the damage rates of classified assets at different level of water depth should be properly modified according to the duration of inundation and the vulnerabilities of the assets in the future.
3. The flood risk is expressed as R-D function, which contains probability and consequence of flood. The 24-h design rainfalls with frequency of 0.001, 0.002, 0.005, 0.01, 0.02, and 0.05 were taken as input data for scenario modeling. The simulation results are utilized as data sample for flood damage function construction. The flood risk reduction is expressed as the area between the two curves. The S-shaped R-D function can describe the impact of flood capability well. The result shows that within the flood control capacity, flood risk is reduced by 15.59% and flood risk reduction is significant. Once flood magnitude exceeds the flood control standard, the flood damage will increase sharply. The flood risk is only reduced by 7.06%. It means that the flood prevention measures may cease to be effective when the flood scale exceeds the flood control standard. It is difficult to meet the increasing demand for flood control solely relying on structural measures.
4. *Rc* (critical return period) is associated with the integrated flood defense capability or flood control standard of the study area. Resistance is related to the system’s ability to prevent floods, while resilience determines the ease with which the system recovers from floods [32]. Within the defense standard, the flood control works operate well. Structural measures are taken as the main resistance strategies, which are aimed at flood prevention. When the flood scale exceeds the defense capability, the situation gets out of control. The non-structural measures are taken as the main resilience strategies to minimize the flood impacts and enhance the recovery from those impacts. Under the impact of climate changes and urbanization, the flood risk increases and the flood mitigation become more difficult, complex and long-term. More emphasis should be put on flood forecasting, flood emergency planning and response, and post-flood recovery. A reasonable flood risk analysis is important, which can be utilized for land use planning, for flood control works design, and for emergency response decision making.

## Figures and Tables

**Table 1:** The reference value of roughness for different land uses.

Land Use	Thicket	Dry Farmland	Paddy Field	Open Space
Roughness	0.065	0.035	0.035	0.025–0.035

**Table 2:** The flood damage based on survey data of Typhoon Fitow (million CNY).

Water Conservancy Facilities	Agriculture	Industry and Transportation	Other	Total
24.00	69.34	21.43	17.35	132.12

**Table 3:** The flood damage simulation results (million CNY).

**Residential Building Losses**	**Residential Property Losses**	**Agriculture Losses**	**Industry Losses**	**Industrial Production Losses**
6.21	17.31	71.26	18.85	9.98
**Business Assets Losses**	**Business Revenue Losses**	**Highway Losses**	**Railway Losses**	**Direct Economic Losses**
1.46	2.41	2.66	0.46	130.60

**Table 4:** The simulation results of inundation.

Statistical Index	Return Period (Year)	24-h Areal Precipitation (mm)	The Largest Inundation Area (km^2^)	The Maximum Water Depth (m)	Average Water Depth (m)	Inundation Area of Water Depth ≥0.5 m (km^2^)
**Condition 1**	5	134.5	1287.75	1.09	0.12	3.71
	10	169.1	1602.62	1.47	0.13	8.93
	20	203.1	1805.49	1.79	0.15	16.74
	50	247.5	1984.76	2.01	0.17	32.84
	100	280.8	2083.45	2.11	0.19	49.35
	200	313.9	2161.28	2.19	0.20	70.81
	1000	390.3	2290.87	2.37	0.24	137.87
**Condition 2**	5	134.5	1520.35	1.27	0.12	2.88
	10	169.1	1752.93	1.62	0.14	8.65
	20	203.1	1906.28	1.90	0.15	16.66
	50	247.5	2046.95	2.07	0.18	33.20
	100	280.8	2123.97	2.15	0.19	50.40
	200	313.9	2187.96	2.22	0.21	72.49
	1000	390.3	2299.33	2.40	0.25	143.70

**Table 5:** Normalization thresholds of the flood depth.

Grade	Water Depth	Impact	Hazard
I	0 m < h ≤ 0.15 m	The curb is usually 15 cm, thus it has hardly effect on production and life.	None
II	0.15 m < h ≤ 0.30 m	It can be able to invade simpler house, with doors-still next to the level of the sidewalk.	Low
III	0.30 m < h ≤ 0.50 m	It can interrupt traffic of vehicles and mainly of people.	Medium
IV	h > 0.5 m	The water will very probably have already invaded the interior of houses, causing damages to their structure and content.	High

**Table 6:** The simulation results of flood damage (million CNY).

Statistical Index	Return Period	Building	Property	Agriculture	Industry	Commerce	Road	Railway	Total
**Condition 1**	5	49.74	84.60	104.25	104.86	14.55	5.44	0.22	363.66
	10	103.86	165.69	207.69	296.52	36.19	14.12	0.79	824.85
	20	172.77	327.59	512.68	452.13	55.95	20.71	1.15	1542.99
	50	309.73	515.66	716.81	702.35	89.18	31.29	1.86	2366.87
	100	452.96	694.48	877.44	929.16	120.21	39.90	2.41	3116.57
	200	615.90	888.56	1022.86	1181.11	155.18	48.82	3.04	3915.47
	1000	1086.34	1409.14	1331.91	1841.77	249.47	75.76	4.54	5998.94
**Condition 2**	5	53.54	97.92	160.41	141.66	19.74	6.02	0.29	479.57
	10	118.41	193.09	277.38	265.99	36.45	10.99	0.55	902.86
	20	199.39	376.88	613.29	539.47	67.43	23.40	1.26	1821.13
	50	350.30	574.33	823.56	802.45	103.94	33.80	1.88	2690.26
	100	547.43	831.52	106.224	1132.48	150.15	46.20	2.80	3772.82
	200	673.53	958.04	1104.33	1283.01	171.36	51.99	3.07	4749.18
	1000	1154.46	1483.33	1389.57	1945.60	269.95	77.84	4.46	6325.21

**Table 7:** Fitting result of two functions.

Index	Function	*A*	*R_c_*	*k*	Adjusted *R*^2^
**Condition 1**	Gompertz	5859.27	66.00	0.0092	0.92
	Logistic	5857.20	115.18	0.013	0.89
**Condition 2**	Gompertz	6047.61	47.97	0.013	0.94
	Logistic	5886.97	76.52	0.019	0.91
